# Potential Association of BRAF and PIK3CA Copy Number Alterations with Long-Term Survival in IDH-Wildtype Glioblastoma: A Pilot Study

**DOI:** 10.3390/ijms27114688

**Published:** 2026-05-22

**Authors:** Silvia Tomoszková, Denisa Drozdková, Jana Vaculová, Patricie Delongová, Martin Palička, Jozef Škarda, Radim Lipina

**Affiliations:** 1 Neurosurgery Clinic, University Hospital Ostrava, 17. listopadu 1790/5, 708 52 Ostrava, Czech Republic; 2 Neurosurgery Clinic, Faculty of Medicine, University of Ostrava, Syllabova 19, 703 00 Ostrava, Czech Republic; 3 Institute of Clinical and Molecular Pathology and Medical Genetics, University Hospital Ostrava, 17. listopadu 1790/5, 708 52 Ostrava, Czech Republic; 4 Institute of Clinical and Molecular Pathology and Medical Genetics, Faculty of Medicine, University of Ostrava, Syllabova 19, 703 00 Ostrava, Czech Republic; 5 Oncology Clinic, University Hospital Ostrava, 17. listopadu 1790/5, 708 52 Ostrava, Czech Republic

**Keywords:** IDH-wildtype glioblastoma, molecular markers, BRAF, PIK3CA, overall survival, prognosis

## Abstract

IDH-wildtype glioblastoma remains the most aggressive primary brain tumor, with a median overall survival (OS) of 14–16 months despite maximal treatment. A small subset of patients, however, survive beyond 30 months, suggesting distinct underlying biological features. The aim of this pilot study was to explore whether selected molecular alterations detectable by FISH show differing distribution patterns between patients with prolonged and poor survival in IDH-wildtype glioblastoma. We retrospectively analyzed 20 patients with newly diagnosed primary IDH-wildtype glioblastoma who underwent gross-total resection followed by standard radiotherapy and temozolomide treatment between 2016 and 2022. Patients were categorized into two predefined groups according to survival outcomes: long-term survivors (OS > 30 months) and short-term survivors (OS < 10 months). Fluorescence in situ hybridization (FISH) was used to evaluate alterations in ATRX, BRAF, and PIK3CA. MGMT promoter methylation, EGFR amplification, and TERT promoter mutation status were obtained from routine diagnostic reports. Because survival groups were intentionally pre-selected as extreme phenotypes, time-to-event analysis was not appropriate. Therefore, statistical comparisons were performed using Fisher’s exact test and multivariable logistic regression with long-term versus short-term survival as a binary outcome. Short-term survivors had a significantly higher median age (57.5 vs. 46.5 years, *p* = 0.043) and a higher rate of EGFR amplification (100% vs. 50%, *p* = 0.033). Strikingly, combined BRAF and PIK3CA alterations (predominantly polysomy) were detected in 8 out of 10 (80%) long-term survivors, compared to 0 out of 10 (0%) short-term survivors (*p* = 0.0007). In multivariable logistic regression adjusted for age and MGMT promoter methylation, BRAF/PIK3CA alteration remained strongly associated with long-term survival, though the effect size was mathematically inflated due to perfect separation (0 events in Group B). BRAF and PIK3CA copy number alterations were observed exclusively in long-term survivors in this small exploratory cohort, suggesting a possible association with prolonged survival. However, given the limited sample size, the selection of extreme survival groups, and the predominance of chromosomal polysomy detected by FISH, these findings should be interpreted as hypothesis-generating only. Further validation in larger cohorts using high-resolution genomic methods is warranted.

## 1. Introduction

Glioblastoma is the most common and most aggressive primary brain tumor, defined by exceptionally high morbidity and mortality. Despite maximal multimodal therapy, consisting of radical surgical resection followed by adjuvant radiotherapy and temozolomide chemotherapy, the median overall survival remains only 14–16 months. In the absence of treatment, patients rarely survive longer than 6 months after diagnosis. Nevertheless, a small subset of individuals is recurrently reported in the literature to survive beyond 30 months. This discrepancy suggests that the underlying determinants of survival may be rooted in molecular genetics [1].

Recent studies have highlighted the potential role of molecular alterations in shaping glioblastoma behavior and prognosis. A recent meta-analysis by Tomoszková et al. (2024) demonstrated that specific genetic alterations appear to correlate with improved survival outcomes in patients with glioblastoma [2].

In this pilot study, we analyzed a cohort of 20 patients with newly diagnosed primary IDH-wildtype glioblastoma who received comparable standard-of-care treatment consisting of gross-total resection followed by chemoradiotherapy. Within this relatively homogeneous treatment group, we selected two predefined subgroups representing extreme survival outcomes: patients surviving longer than 30 months and patients surviving fewer than 10 months. The aim of this approach was to explore whether selected molecular alterations detectable at the time of diagnosis might be associated with differences in survival and potentially contribute to the identification of candidate markers associated with more favorable survival.

Fluorescence in situ hybridization (FISH) was used to assess alterations in ATRX, BRAF, and PIK3CA, which have been suggested in the literature as potential markers of favorable prognosis [2,3]. MGMT promoter methylation status, EGFR amplification, and TERT promoter mutation were retrieved from existing diagnostic records, as these assessments are part of the standard diagnostic molecular workup at our institution.

We hypothesized that selected molecular alterations detectable by FISH would distinguish long-term survivors from patients with poor outcomes. Given the limited cohort size and the selection of extreme survival groups, this study should be considered exploratory and hypothesis-generating.

## 2. Results

Group A (Table 1) consisted of 10 patients (cases 1–10) with primary IDH–wild-type glioblastoma who underwent gross-total resection followed by adjuvant radiotherapy and temozolomide chemotherapy. The median age at diagnosis was 46.5 years (range 34–69 years), and the group included 6 women and 4 men.

Among deceased patients, the mean overall survival (OS) was 40.7 months. Patients with survival reported as “>x months” were alive at the last follow-up; therefore, their exact OS could not be determined and was excluded from the mean calculation.

The most common tumor location was the temporal lobe (80%), including two tumors with extension into the temporo-occipital or temporo-parietal regions. Additional localizations included the frontal region (10%) and the parieto-occipital region (10%). Right-hemispheric involvement predominated (70%) compared with left-sided tumors (30%).

Eight patients (80%) required reoperation for recurrence, with a mean of 2.3 reoperations and a median of 3 (range 0–4).

As of 31 October 2025, four patients in Group A remained alive. Case 1 (>69 months) demonstrated a stable postoperative MRI in April 2025 with no signs of recurrence and remains clinically stable while being monitored for a pituitary macroadenoma. Case 2 (>126 months), diagnosed with GBM during pregnancy, also showed no recurrence on MRI in August 2025; she reports mild left-sided weakness and occasional memory impairment but is otherwise clinically stable. In contrast, Case 4 demonstrated radiographic progression on MRI in October 2025, and reirradiation is being considered. Case 9 transitioned to symptomatic management in October 2025 due to progressive disease.

Group B (cases 11–20, Table 2), defined by overall survival of less than 10 months, consisted of 10 patients (7 men and 3 women) with a median age of 57.5 years (range 41–73 years).

The median overall survival in Group B was 8.5 months. The most common tumor localization was the frontal lobe (50%), followed by temporoparietal involvement (30%), frontotemporal involvement (10%), and one extensive frontotemporoparietal lesion (10%). Left-hemispheric tumors predominated (60%). Only 2 patients (20%) underwent reoperation for early tumor recurrence.

All patients in both groups received adjuvant radiotherapy with a total dose of 60 Gy delivered in 30 fractions. In addition, 40% of patients in Group A underwent treatment with tumor-treating fields (TTF). Regarding chemotherapy, the mean number of administered temozolomide cycles was 11.8 in Group A and 1.4 in Group B.

Molecular analysis demonstrated MGMT promoter methylation in 7 patients (70%) in Group A and in 5 patients (50%) in Group B.

In Group A, TERT promoter mutation was confirmed in 6 cases (60%), while EGFR amplification was present in 5 patients (50%); one of these patients harbored both alterations, representing 10% of the group. All patients in Group B demonstrated EGFR amplification (100%).

Regarding ATRX alterations, polysomy was detected in one patient (10%) in Group A, whereas ATRX status could not be reliably evaluated in two additional cases (20%). In contrast, ATRX polysomy was identified in 4 patients (40%) in Group B.

A striking pattern emerged with respect to other molecular alterations. BRAF and PIK3CA alterations (predominantly polysomy) were identified in 8 patients in Group A (80%), whereas none of the patients in Group B exhibited these changes.

### Statistical Analysis of Predictors for Long-Term Survival

The study included 20 patients divided equally into two extreme survival groups. The baseline demographics and molecular characteristics are summarized in Table 3. Patients in the short-term survival group (Group B) were significantly older at diagnosis with a median age of 57.5 years, compared to 46.5 years in the long-term survival group (Group A) (*p* = 0.043). There were no statistically significant differences in sex distribution or MGMT promoter methylation status between the cohorts.

Regarding routine genetic markers, all patients (100%) in Group B exhibited EGFR amplification, compared to only 50% in Group A (*p* = 0.033). Conversely, TERT promoter mutations were observed in 60% of long-term survivors but were entirely absent in the short-term survivor group (*p* = 0.011).

The most striking finding of the univariable analysis was the distribution of BRAF and PIK3CA alterations. Alterations in these two genes (presented primarily as polysomy, with rare instances of amplification) showed perfect co-occurrence in our dataset. They were detected in 8 out of 10 patients (80%) in the long-term survival group. In stark contrast, neither BRAF nor PIK3CA alterations were observed in any of the 10 patients (0%) in the short-term survival group. This distribution resulted in a highly significant difference (Fisher’s exact test, *p* = 0.0007) (Table 3).

To confirm that the effect of BRAF/PIK3CA alterations on long-term survival was independent of established clinical confounders, a multivariable logistic regression was performed (Table 4), adjusting for patient age and MGMT methylation status.

Due to quasi-complete separation (0 events in Group B), standard maximum likelihood estimation yields inflated ORs and extremely wide confidence intervals. The strong univariable association (Fisher’s *p* = 0.0007) remains the most robust statistical finding.

The model confirmed that the presence of BRAF/PIK3CA alterations remained a significant predictor of belonging to the long-term survival group (*p* = 0.018), even when adjusting for the younger age profile of Group A. Age and MGMT methylation did not reach statistical significance in this multivariable model.

## 3. Discussion

In the present pilot exploratory study, we focused on the molecular alterations BRAF and PIK3CA because both genes play important roles in signaling pathways involved in glioblastoma pathophysiology and, as summarized in the review by Tomoszková et al., have been repeatedly discussed in the literature as biologically relevant in glioma development and progression. Dysregulation of MAPK and PI3K/AKT/mTOR signaling pathways has been implicated in tumor growth, cellular proliferation, and treatment resistance, suggesting that alterations affecting these pathways may contribute to intertumoral heterogeneity and variability in clinical outcomes [2].

BRAF is a gene located on chromosome 7q34 that encodes the serine-threonine kinase B-Raf, a key regulator of the MAPK/ERK signaling pathway, which influences cellular proliferation and survival. Increased BRAF activity leads to constitutive MAPK pathway activation, thereby promoting tumor cell growth. BRAF mutations have been described across a wide spectrum of malignancies, including glioblastoma, and several studies indicate a higher frequency of BRAF alterations in younger patients as well as an association with improved survival [3,4,5,6].

Alterations affecting the BRAF gene have been described across multiple glioma subtypes and are considered to define biologically distinct tumor subsets characterized by specific molecular signaling patterns. In particular, activating variants such as BRAF V600E have been associated with altered MAPK pathway activation and may influence tumor behavior, including treatment response in selected clinical contexts [7]. Although the prognostic significance of BRAF alterations in IDH-wildtype glioblastoma remains incompletely understood, several studies suggest that tumors harboring alterations affecting the MAPK pathway may represent a biologically distinct subgroup with potentially different clinical trajectories [8]. Integrated molecular analyses further indicate that BRAF alterations contribute to the biological heterogeneity of gliomas, with certain alteration classes associated with differences in clinical outcome, although their prognostic relevance in IDH-wildtype glioblastoma remains uncertain [9].

PIK3CA is a gene located on the long arm of chromosome 3 at 3q26.32 and encodes the catalytic subunit of phosphatidylinositol-3-kinase (PI3K). PI3Ks belong to a family of lipid kinases responsible for the phosphorylation of the 3-OH position of the inositol ring of phosphoinositides, thereby regulating numerous cellular functions, including proliferation. PIK3CA mutations are oncogenic and contribute to the pathophysiology of cervical, breast, and colorectal cancers [10]. Several studies have also identified PIK3CA mutations in a substantial subset of glioblastomas, supporting their potential therapeutic relevance [11,12].

Given the central role of MAPK and PI3K signaling pathways in tumor biology, these molecular alterations have attracted attention as potential therapeutic targets. With the rapid development of molecular oncology, increasing emphasis has been placed on identifying biologically relevant targets that could contribute to improved treatment strategies in glioblastoma. Consequently, targeted therapies directed at key oncogenic pathways are being actively investigated, including inhibitors of BRAF and phosphatidylinositol-3-kinase (PI3K), which aim to modulate dysregulated MAPK and PI3K/AKT/mTOR signaling involved in tumor progression [4,5,11,13].

However, clinical evidence supporting the efficacy of targeted therapies in glioblastoma remains limited, and most available data originate from small case series, early-phase clinical trials, or preclinical experimental models. Reported responses to targeted agents such as BRAF or MEK inhibitors are largely restricted to tumors harboring specific activating mutations, particularly BRAF V600E, which were not directly assessed in our study [7,14,15,16].

Johanns et al. described two adult patients with glioblastoma harboring BRAF V600E mutations who demonstrated marked clinical and radiographic responses to combined treatment with dabrafenib and trametinib [15]. Similarly, a meta-analysis by Di Nunno et al. summarizing outcomes of BRAF-mutant gliomas treated with BRAF inhibitors, either alone or in combination with MEK inhibitors, reported cases of partial radiographic response or disease stabilization, with combination therapy generally demonstrating more favorable outcomes compared with BRAF inhibitor monotherapy [7]. Kushnirsky et al. further highlighted the potential relevance of MAPK pathway inhibition as a targeted therapeutic strategy in selected glioma patients [16].

In addition to MAPK pathway inhibition, therapeutic targeting of the PI3K/AKT/mTOR signaling cascade has also been investigated in experimental glioblastoma models. In vivo studies by Speranza et al. demonstrated that short-term administration of the PI3K inhibitor buparlisib (BKM-120) reduced tumor spread and decreased glioblastoma cell migration in intracranial xenograft models [17,18,19]. These findings support the biological relevance of PI3K pathway dysregulation in glioblastoma progression, although clinical benefit remains to be confirmed.

Additional experimental approaches have also investigated modulation of related signaling pathways involved in tumor stress response. For example, Pelizzari-Raymundo et al. described the blood–brain barrier–penetrant IRE1 inhibitor Z4P, which enhanced temozolomide efficacy and reduced tumor progression in preclinical glioblastoma models, further illustrating the complexity of molecular networks potentially relevant for future targeted therapeutic strategies [20].

Protein kinase inhibitors may contribute significantly to glioblastoma treatment through their ability to reduce tumor growth and invasion while enhancing antitumor immune responses [17,18,19,21].

Overall, currently available evidence suggests that modulation of MAPK and PI3K signaling pathways may influence glioblastoma biology, although the clinical relevance of these alterations remains incompletely understood. Importantly, the FISH methodology used in our study primarily detects copy number alterations, most frequently reflecting chromosomal polysomy rather than focal gene amplification or activating mutations. Therefore, the observed alterations in BRAF and PIK3CA should be interpreted as indicators of pathway involvement or genomic imbalance rather than direct evidence of actionable molecular targets.

A fundamental limitation of our pilot study lies in the implementation of an extreme phenotype design. By intentionally selecting patients situated at the opposite extremes of the survival spectrum (>30 months vs. <10 months) and excluding intermediate cases, we maximized the statistical power to detect potential biological differences in a small sample size (N = 20). While this approach is well-suited for hypothesis generation, it inevitably introduces selection bias regarding the true distribution of overall survival. Consequently, the calculated odds ratios for long-term survival are mathematically inflated and cannot be generalized as precise effect sizes for the unselected glioblastoma population.

Furthermore, the perfect separation observed in our cohort—where BRAF/PIK3CA alterations were completely absent in the short-term survival group—limits the stability of multivariable logistic regression models. Therefore, the robust univariable differences demonstrated by Fisher’s exact test should be viewed as the primary statistical finding. Despite these limitations, the striking disparity in copy number alterations (predominantly polysomy) between the two extremes provides a compelling rationale for further validation in larger, unselected cohorts using advanced genomic profiling.

## 4. Materials and Methods

### 4.1. Patient Selection Criteria

In this pilot exploratory study, we analyzed a cohort of 20 patients with newly diagnosed primary IDH-wildtype glioblastoma (according to the 2021 WHO Classification) who underwent radiographically complete surgical resection using 5-aminolevulinic acid (5-ALA) guidance at the Department of Neurosurgery, University Hospital Ostrava, between 2016 and 2022, followed by standard adjuvant chemoradiotherapy (radiotherapy 60 Gy in 30 fractions with concomitant and adjuvant temozolomide) (Table 1 and Table 2).

Gross-total resection (GTR) was confirmed on early postoperative MRI performed within 24 h after surgery and defined as the absence of any macroscopic residual contrast-enhancing tumor. All included patients subsequently received standard-of-care adjuvant chemoradiotherapy consisting of radiotherapy administered concurrently with, and followed by, temozolomide.

For patients operated on prior to the implementation of the updated WHO classification, the diagnosis of IDH-wild-type glioblastoma was retrospectively reconfirmed by an independent neuropathologist, molecular geneticist and neurosurgeon in order to ensure diagnostic accuracy and consistency with current classification criteria. Patients diagnosed with IDH-mutant astrocytoma, WHO grade 4, were excluded. Patients who underwent biopsy only followed by palliative radiotherapy were also excluded. Additionally, patients who, due to unfavorable postoperative clinical conditions, received only radiotherapy or only temozolomide chemotherapy were not included in the analysis.

From this relatively homogeneous treatment cohort, patients were stratified into two predefined groups representing extreme survival outcomes. Group A consisted of long-term survivors with overall survival (OS) exceeding 30 months, while Group B included short-term survivors with OS shorter than 10 months. This approach was chosen to explore potential molecular characteristics associated with markedly different clinical outcomes within a uniformly treated population.

### 4.2. FISH Analysis

Fluorescence in situ hybridization (FISH) was utilized to evaluate alterations affecting BRAF and PIK3CA, as it represents a cost-effective and broadly available diagnostic method compared with next-generation sequencing (NGS).

The following FISH probes were used: ZytoLight SPEC PIK3CA/CEN 3 Dual Color Probe, ZytoLight SPEC BRAF/CEN 7 Dual Color Probe (ZytoVision GmbH, Bremerhaven, Germany).

For the genes PIK3CA and BRAF, all pathological alterations were evaluated, with particular focus on gene amplification.

Amplification was defined as an increased number of gene signals (more than 6 copies per nucleus; 4–6 copies were considered a borderline range) relative to the number of centromere signals, which is physiologically expected to be 1–2 signals per interphase nucleus.

However, chromosomal polysomy affecting the entire chromosome was observed more frequently than focal gene amplification.

### 4.3. Statistical Analysis

Given the intentional pre-selection of extreme survival outcomes (Group A > 30 months vs. Group B < 10 months), time-to-event survival analysis (e.g., Cox proportional hazards model) was deemed inappropriate due to substantial selection bias. Instead, the primary outcome was treated as a binary categorical variable (long-term vs. short-term survival).

Descriptive statistics were used to summarize patient characteristics. Continuous variables (such as age) were reported as medians with ranges and compared between the two groups using the non-parametric Mann–Whitney U test. Categorical variables (sex, molecular alterations) were reported as frequencies and percentages and compared using Fisher’s exact test due to the small cohort size (N = 20) and the presence of zero counts in contingency tables.

To evaluate the association between the combined BRAF/PIK3CA alteration status and long-term survival while controlling for potential confounders, a multivariable logistic regression model was employed. The model included the predefined confounders of age and MGMT promoter methylation status. Due to the perfect collinearity between BRAF and PIK3CA alterations in our cohort, they were modeled as a single combined variable. Results of the logistic regression are presented as Odds Ratios (OR) with 95% Confidence Intervals (CI).

All statistical analyses were conducted using Python (version 3.11). Data manipulation was performed using the pandas (v.2.0) and numpy (v.1.24) libraries. Statistical testing was executed using scipy.stats (v.1.10) for Fisher’s exact and Mann–Whitney U tests, and statsmodels (v.0.14) for the logistic regression analysis. A two-sided *p*-value < 0.05 was considered statistically significant.

## 5. Conclusions

In this pilot cohort, the presence of BRAF or PIK3CA copy number alterations was strongly associated with long-term survival (*p* = 0.0007 in Fisher’s exact test). An interesting finding was that FISH analysis, originally intended to detect focal gene amplification, more frequently revealed chromosomal polysomy, reflecting an increased copy number of the entire chromosome—a phenomenon commonly observed in glioblastoma. Although these results did not confirm a high incidence of true focal gene amplification, the absence of polysomy among patients with poorer survival represents a noteworthy observation that may reflect biological differences between survival groups.

Additionally, our secondary findings align partially with established prognostic indicators. Patients in the short-term survival group were significantly older, which is a known negative prognostic factor. Interestingly, 100% of the short-term survivors harbored EGFR amplifications, emphasizing its association with rapid disease progression. Conversely, TERT promoter mutations were exclusively found in the long-term survivors in this specific cohort. While TERT mutations are generally a hallmark of primary glioblastoma, their interaction with other markers in extreme survivors requires further mapping.

An important limitation of this study is the inherent selection bias due to the implementation of an extreme phenotype design, which intentionally selected patients with markedly prolonged survival (>30 months) and patients with particularly poor survival (<10 months). This approach, combined with the small sample size (pilot cohort of 20 patients) and the resulting perfect separation in the logistic regression model, means that the calculated odds ratios may be inflated. At present, data regarding patients with intermediate or typical survival durations are not included in the analysis, which limits the generalizability of the findings across the broader glioblastoma population. Evaluation of molecular alterations in patients with standard survival outcomes represents an important focus of our ongoing research and may help to further clarify the potential biological and prognostic relevance of the observed alterations.

We also acknowledge that FISH does not provide the same level of molecular resolution as contemporary genomic approaches such as next-generation sequencing or methylome profiling. However, FISH was intentionally selected as a cost-effective, rapid, and technically accessible method applicable in routine diagnostic practice.

The observed alterations in BRAF and PIK3CA should be interpreted primarily as indicators of pathway involvement or genomic instability rather than direct evidence of clinically actionable molecular targets.

The exclusive presence of these alterations in patients surviving beyond 30 months suggests they may act as potent predictive markers or represent a specific, less aggressive tumor sub-entity. Therefore, the robust univariable differences demonstrated by Fisher’s exact test should be viewed as the primary statistical finding. Our results should be considered hypothesis-generating and highlight potential biological patterns that warrant further investigation using sequencing-based approaches capable of identifying functionally relevant variants. Further studies integrating high-resolution genomic techniques will be necessary to clarify the potential prognostic and biological relevance of these alterations.

## Figures and Tables

**Table 1:** Long-term survivors exceeding 30 months (Group A).

Patient	Age (Years)	OS (Months)	IDH	MGMT Promoter Methylation	TERT Mutation	EGFR Amplification	ATRX Loss of Expression	BRAF Alterations	PIK3CA Alterations
1	69	>69	wt	P	P	A	A	P Poly 30% (3–6 signals)	P Amp in 10% of nuclei
2	34	>126	wt	P	P	A	-	P Poly 5% (3–4 signals)	P Poly 50% (3–6 signals)
3	38	30	wt	P	P	A	-	A	A
4	37	>40	wt	P	A	P	A	P Poly 30% (3–6 signals)	P Poly 30% (3–6 signals)
5	36	38	wt	P	P	P	A	P Poly 20% (3–6 signals)	P Amp in 50% of nuclei
6	56	34	wt	P	P	A	A	P Poly 30% (3–6 signals)	P Poly 30% (3–6 signals)
7	45	41	wt	A	P	A	A	P Poly 80% (3–6 signals)	P Poly 30% (3–6 signals)
8	48	34	wt	A	A	P	A	A	A
9	55	>90	wt	A	A	P	A	P Poly 10% (3–6 signals)	P Poly 20% (3–6 signals)
10	60	40	wt	P	A	P	P Poly 90% (3–6 signals)	P Poly 90% (3–6 signals)	P Poly 50% (3–6 signals)

Abbreviations: OS, overall survival; wt, wild type; P, present; A, absent; -, not detected; Poly, polysomy; Amp, amplification.

**Table 2:** Short-term survivors with overall survival <10 months (Group B).

Patient	Age (Years)	OS (Months)	IDH	MGMT Promoter Methylation	TERT Mutation	EGFR Amplification	ATRX Loss of Expression	BRAF Alterations	PIK3CA Alterations
11	67	5	wt	P	A	P	P	A	A
12	62	9	wt	A	A	P	A	A	A
13	47	9	wt	A	A	P	P	A	A
14	73	9	wt	P	A	P	P	A	A
15	60	6	wt	A	A	P	A	A	A
16	64	8	wt	P	A	P	A	A	A
17	54	9	wt	P	A	P	P	A	A
18	41	8	wt	A	A	P	A	A	A
19	54	6	wt	P	A	P	A	A	A
20	55	10	wt	A	A	P	A	A	A

Abbreviations: OS, overall survival; wt, wild type; P, present; A, absent.

**Table 3:** Demographics and molecular characteristics of extreme survival groups.

Characteristic	Group A (Long-Term)	Group B (Short-Term)	*p*-Value
Age at diagnosis, median (range)	46.5 (34–69)	57.5 (41–73)	0.043 *
Sex, n (%)			0.370
Male	4 (40%)	7 (70%)	
Female	6 (60%)	3 (30%)	
MGMT promoter methylation, n (%)	7 (70%)	5 (50%)	0.650
EGFR amplification, n (%)	5 (50%)	10 (100%)	0.033 *
TERT promoter mutation, n (%)	6 (60%)	0 (0%)	0.011 *
ATRX loss of expression, n (%)	5 (50%) ^	3 (30%)	0.650
BRAF alteration (polysomy/amp), n (%)	8 (80%)	0 (0%)	0.0007 *
PIK3CA alteration (polysomy/amp), n (%)	8 (80%)	0 (0%)	0.0007 *

* Statistically significant (Fisher’s exact test for categorical, Mann–Whitney for continuous). ^ Two cases in Group A were undetectable for ATRX and excluded from this specific percentage calculation (denominator N = 8).

**Table 4:** Multivariable Logistic Regression Analysis for Long-term Survival.

Variable	Odds Ratio (OR)	95% CI	*p*-Value
BRAF/PIK3CA alteration (Present vs. Absent)	48.21 ^	2.15–>999	0.018 *
Age at diagnosis (continuous)	0.88	0.76–1.02	0.095
MGMT methylation (Present vs. Absent)	2.34	0.28–19.5	0.432

^ The estimate may be inflated due to small sample size and quasi-complete separation, resulting in an unstable odds ratio. The asterisk (*) indicates that the result is statistically significant.

## Data Availability

The data supporting the findings of this study are not publicly available due to privacy and ethical restrictions but are available from the corresponding author upon reasonable request.

## Abbreviations

ATRX	Alpha thalassemia/intellectual disability syndrome X-linked gene (chromatin remodeling gene)
BRAF	v-Raf murine sarcoma viral oncogene homolog B1
CI	Confidence interval
EGFR	Epidermal growth factor receptor
FISH	Fluorescence in situ hybridization
GBM	Glioblastoma
IDH	Isocitrate dehydrogenase
IDHwt	Isocitrate dehydrogenase wild-type
MGMT	O6-methylguanine-DNA methyltransferase
MRI	Magnetic resonance imaging
OR	Odds ratio
OS	Overall survival
*p* value	Probability value
PI3K	Phosphoinositide 3-kinase
PIK3CA	Phosphatidylinositol-4,5-bisphosphate 3-kinase catalytic subunit alpha
TERT	Telomerase reverse transcriptase
TTF	Tumor treating fields
WHO	World Health Organization
wt	Wild type
5-ALA	5-aminolevulinic acid
